# Evolution of Esophagectomy for Cancer Over 30 Years: Changes in Presentation, Management and Outcomes

**DOI:** 10.1245/s10434-020-09200-3

**Published:** 2020-10-18

**Authors:** S. Michael Griffin, Rhys Jones, Sivesh Kathir Kamarajah, Maziar Navidi, Shajahan Wahed, Arul Immanuel, Nick Hayes, Alexander W. Phillips

**Affiliations:** 1 Northern Oesophagogastric Unit, Royal Victoria Infirmary, Newcastle upon Tyne NHS Hospitals, Newcastle-upon-Tyne, UK; 2 School of Medical Education, Newcastle University, Newcastle-upon-Tyne, UK

## Abstract

**Background:**

Esophageal cancer has seen a considerable change in management and outcomes over the last 30 years. Historically, the overall prognosis has been regarded as poor; however, the use of multimodal treatment and the integration of enhanced recovery pathways have improved short- and long-term outcomes.

**Objective:**

The aim of this study was to evaluate the changing trends in presentation, management, and outcomes for patients undergoing surgical treatment for esophageal cancer over 30 years from a single-center, high-volume unit in the UK.

**Patients and Methods:**

Data from consecutive patients undergoing esophagectomy for cancer (adenocarcinoma or squamous cell carcinoma) between 1989 and 2018 from a single-center, high-volume unit were reviewed. Presentation method, management strategies, and outcomes were evaluated. Patients were grouped into successive 5-year cohorts for comparison and evaluation of changing trends.

**Results:**

Between 1989 and 2018, 1486 patients underwent esophagectomy for cancer. Median age was 65 years (interquartile range [IQR] 59–71) and 1105 (75%) patients were male. Adenocarcinoma constituted 1105 (75%) patients, and overall median survival was 29 months (IQR 15–68). Patient presentation changed, with epigastric discomfort now the most common presentation (70%). An improvement in mortality from 5 to 2% (*p* < 0.001) was seen over the time period, and overall survival improved from 22 to 56 months (*p* < 0.001); however, morbidity increased from 54 to 68% (*p* = 0.004).

**Conclusions:**

Long-term outcomes have significantly improved over the 30-year study period. In addition, mortality and length of stay have improved despite an increase in complications. The reasons for this are multifactorial and include the use of perioperative chemo(radio)therapy, the introduction of an enhanced recovery pathway, and improved patient selection.

Esophageal cancer is still widely regarded as a diagnosis with a poor outcome. Globally, it affects approximately 450,000 patients each year.^1^ Reported cure rates in the 1970s in the UK and US were only 4–5%, increasing to 15–18% by 2010.^2^,^3^ Significant changes in the management of esophageal cancer have been introduced over the past two decades. Neoadjuvant treatment is now regarded as the gold standard for those patients with locally advanced disease, and commonly involves the administration of chemotherapy or chemoradiotherapy.^4^–^6^ Furthermore, surgical techniques are evolving with the introduction of endoscopic therapy for early-stage cancers^7^ and minimally invasive and robotic techniques.^8^–^10^ There is also an increasing appreciation of improving the whole patient pathway involving prehabilitation and enhanced recovery in the immediate postoperative setting.^11^,^12^

The overall impact of these changes on survival is unknown. Understanding the impact of interventions and identifying areas where little progress has been made is key to determining which components of treatment need further targeting to try and improve outcomes.

This study evaluates outcomes following esophageal resection over the last 30 years in a single-center, high-volume unit in the UK. Key changes in management strategy and their potential impact have been highlighted. Our aim was to identify changes in patient management and outcomes.

## Patients and Methods

### Patient Population

A contemporaneously maintained database of all patients with carcinoma of the esophagus or gastroesophageal junction between 1989 and 2018 was reviewed. Patients were discussed by the multidisciplinary team (MDT) and underwent staging investigations that evolved over the time period of this study. Patients who underwent esophagectomy with curative intent for cancer of the esophagus or gastroesophageal junction were included.

### Staging

Initial staging comprised endoscopy with biopsy, endoscopic ultrasonography, and a thoracoabdominal computed tomography (CT) scan. A positron emission tomography (PET [CT]) scan was not part of the initial staging but was increasingly used over the time interval and is now part of routine care. In the early part of the study period, bone scans were employed to help determine if there was metastatic disease. Staging laparoscopy with washings for cytology was employed where there was an abdominal component to the disease, and neck ultrasound and fine needle aspiration (FNA) were employed where concerns about neck pathology existed. Staging was updated according to the TNM 8th edition.^13^

### Treatment

During the early part of the study period, patients with proven cancer were offered transthoracic esophagectomy as a unimodality treatment. As neoadjuvant therapy became the standard of care, those with cancer staged at T2 N0 or earlier, and those with concerns that neoadjuvant treatment would decondition the patient such that they may not receive surgery, were offered unimodality treatment in the form of surgery. Those diagnosed with locally advanced disease (T3+ or N+) were treated with neoadjuvant chemo/radiotherapy followed by surgery.

Multiple neoadjuvant regimens were employed in the present study, determined by the standard of care and recruiting trials at the time of each patient’s treatment. This included cisplatin and fluorouracil as per the OE02 regimen;^6^ epirubicin, cisplatin, and either fluorouracil or capecitabine (ECF/ECX) as per the MAGIC regimen;^5^ and chemoradiotherapy as per the CROSS regimen, as the main neoadjuvant treatments employed.

Transthoracic esophagectomy with two-field lymphadenectomy using a conventional approach as previously reported^14^–^16^ was the most frequently performed procedure. Patients were routinely discharged post-surgery to critical care (high dependency unit or intensive treatment unit).

An enhanced recovery protocol was initially piloted in 2014 and became the standard of care in 2016. This followed the principles of early patient mobilization, earlier enteral nutrition, and a standardized feeding protocol for patients using their feeding jejunostomy, which have always routinely been placed. In addition, the use of epidurals to provide postoperative analgesia have been superseded by multimodal analgesia, including intrathecal diamorphine, local anesthetic catheters into the paravertebral and rectus sheaths, and patient-controlled analgesic opioids postoperatively.^17^ Patients staged as having an early (T1) cancer with a tumor that was amenable to endoscopic mucosal resection (EMR) were offered this as a potentially curative treatment during the latter years of this study. The initial procedure was performed in 2007, however this became an established modality from 2014 onwards. Patients offered this treatment were kept under close surveillance and surgery was offered if recurrence occurred, and subject to staging described above.

### Pathology

Histopathological reporting was carried out by specialist gastrointestinal pathologists using a standardized proforma in line with guidelines produced by the Royal College of Pathologists. This included tumor type and differentiation, depth of tumor infiltration, and degree of tumor regression according to the Mandard criteria. The total number of lymph nodes and number of nodal metastases from each location were also recorded.^18^ From 1998 onwards, histology specimens were dissected by the operating surgeon into lymph node regions, as has been previously described.^19^

### Follow-Up and Definition of Recurrence

All patients were seen at the outpatient clinic at 3- to 6-month intervals during the first 2 years and every 6 months or annually thereafter, After 5 years, follow-up was on a yearly basis for a total of 10 years. Follow-up was extended until August 2019, ensuring a minimal potential follow-up of 36 months for the evaluation of long-term survival. Recurrence of disease was made on clinical grounds and was confirmed with either CT scans or endoscopically.

### Statistical Analysis

Categorical variables were compared using the Chi square test, and non-normally distributed data were analyzed using the Mann–Whitney U test. Survival was estimated using Kaplan–Meier survival curves and compared using the log-rank test. Multivariable analyses used Cox proportional hazards models (“Appendix 1”). A comparison of outcomes between 5-year periods (1989–1993, 1994–1998, 1999–2003, 2004–2008, 2009–2013, 2014–2018) was also performed. For the final cohort, patients were included up to January 2017 to allow for a minimum 3 years of follow-up. A *p* value < 0.05 was considered to be statistically significant. Data analysis was performed using R Foundation Statistical software (R 3.2.2) with TableOne, ggplot2, Hmisc, Matchit and survival packages (The R Foundation for Statistical Computing, Vienna, Austria), as previously reported.

As a review of past practice and outcomes, this study was deemed exempt from the need for ethical approval.

## Results

### Baseline Demographics

Between 1989 and 2018, 1486 patients underwent esophagectomy for esophageal cancers performed by 12 different surgeons. Median age of the entire cohort was 65 years (interquartile range [IQR] 59–71) and 1105 (75%) patients were male. Adenocarcinoma was the predominant subtype (75%, *n* = 1114 patients), although the proportion changed throughout the 30 years evaluated, from 54% to a maximum of 77% (*p* < 0.001). The majority of patients underwent two-stage, two-field transthoracic esophagectomy (96%, *n* = 1429 patients) and 3% underwent three-stage esophagectomy.

### Patient Presentation

**Fig. 1:**
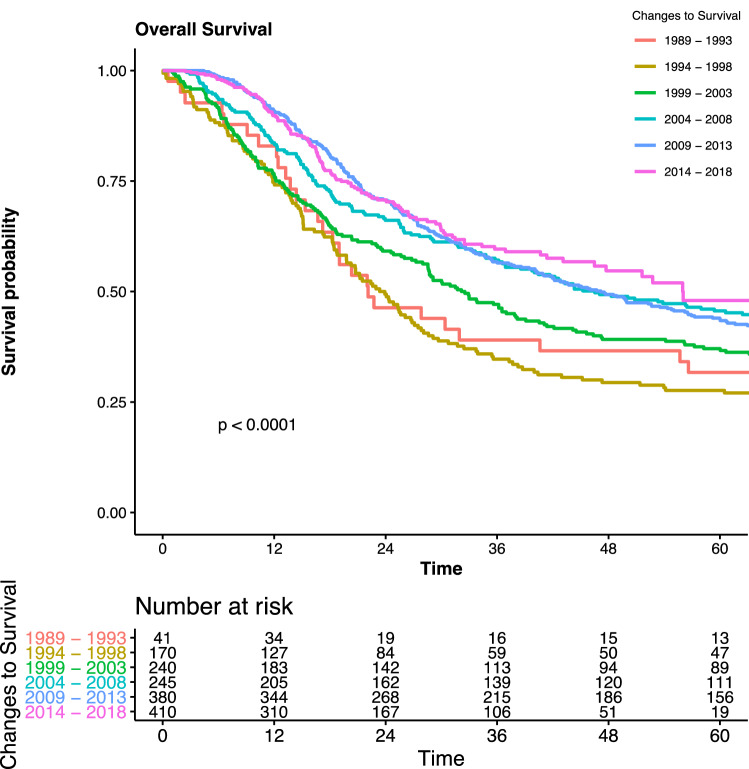
Overall survival, in months, for each time cohort

 Over this 30-year time period, there was an increase in the number of patients undergoing esophagectomy in each 5-year period (Fig. 1). Over the study period, presenting symptoms changed, with a significant fall in the number of patients presenting with weight loss, from 73% in the initial time period to 42% in the final time period (*p* < 0.001). This correlated with a steady increase in the body mass index (BMI) of patients at presentation, from 24 to 27 kg/m^2^ (*p* < 0.001), and fewer patients presenting with anorexia, from 32% in the early time cohort to 5% (*p* < 0.001) [Table 1]. The most common presentation for patients in the most recent cohort was epigastric discomfort, 70% of whom stated it was a symptom, compared with 10–17% in the earliest two cohorts (*p* < 0.001). Patient presentation with regurgitation fell steadily over the years studied, from 56 to 21% (*p* < 0.001), while other ‘red flag’ symptoms or prompts for investigation, such as dysphagia, nausea, reflux, and anemia, fluctuated between cohorts and showed no steady trend.

**Table 1:** Demographics and trends in patient presentation symptoms and lifestyle factors

	Overall	1989–1993	1994–1998	1995–2003	1995–2008	2009–2013	2014–2018	*p* value
*n*	1486	41	170	240	245	380	410	
Age at presentation, years (median [IQR])	65 [59–71]	64 [58–70]	64 [57–70]	65 [56–71]	65 [58–71]	65 [59–72]	66 [60–71]	0.159
Male sex	1105 (75)	29 (71)	130 (76)	169 (70)	180 (73)	277 (73)	320 (78)	0.297
SCC histology	372 (25)	19 (46)	54 (32)	63 (26)	53 (22)	90 (24)	93 (23)	0.004
BMI (median [IQR])	26 [23–29]	24 [21–26]	24 [21–28]	25 [22–28]	26 [24–29]	26 [23–29]	27 [24–30]	< 0.001
Smoking status								< 0.001
Current	371 (25)	19 (46)	61 (36)	79 (33)	58 (24)	74 (19)	80 (20)	
Ex-smoker	649 (44)	10 (24)	63 (37)	97 (40)	110 (45)	186 (49)	183 (45)	
Never	448 (30)	10 (24)	45 (26)	63 (26)	75 (31)	113 (30)	142 (35)	
Unknown	18 (1)	2 (5)	1 (1)	1 (0)	2 (1)	7 (2)	5 (1)	
Alcohol status								< 0.001
Current	1067 (72)	30 (73)	104 (61)	170 (71)	180 (73)	268 (71)	315 (77)	
Ex-drinker	91 (6)	1 (2)	4 (2)	10 (4)	28 (11)	28 (7)	20 (5)	
Never	284 (19)	5 (12)	36 (21)	50 (21)	37 (15)	81 (21)	75 (18)	
Unknown	44 (3)	5 (12)	26 (15)	10 (4)	0 (0)	3 (1)	0 (0)	
ASA grade								< 0.001
1	211 (14)	6 (15)	50 (29)	34 (14)	35 (14)	72 (19)	14 (3)	
2	725 (49)	15 (37)	60 (35)	87 (36)	131 (53)	204 (54)	228 (56)	
3	394 (27)	3 (7)	19 (11)	62 (26)	56 (23)	91 (24)	163 (40)	
4	10 (1)	1 (2)	1 (1)	3 (1)	2 (1)	2 (1)	1 (0)	
Unknown	146 (10)	16 (39)	40 (24)	54 (22)	21 (9)	11 (3)	4 (1)	
Reported weight loss	773 (52)	30 (73)	103 (61)	142 (59)	128 (52)	197 (52)	173 (42)	< 0.001
Anorexia	193 (13)	13 (32)	43 (25)	44 (18)	41 (17)	35 (9)	17 (4)	< 0.001
Epigastric discomfort	610 (41)	4 (10)	29 (17)	73 (30)	99 (40)	120 (32)	285 (70)	< 0.001
Epigastric pain	296 (20)	9 (22)	45 (26)	66 (28)	36 (15)	74 (19)	66 (16)	0.001
Retrosternal pain	313 (21)	10 (24)	29 (17)	70 (29)	72 (29)	76 (20)	56 (14)	< 0.001
Vomiting/regurgitation	501 (34)	23 (56)	87 (51)	103 (43)	85 (35)	115 (30)	88 (21)	< 0.001
Dysphagia								0.449
Unknown	17 (1)	1 (2)	1 (1)	2 (1)	1 (0)	6 (2)	6 (1)	
Can eat normally	404 (27)	4 (10)	37 (22)	68 (28)	77 (31)	102 (27)	116 (28)	
Difficulty with solids	678 (46)	22 (54)	93 (55)	101 (42)	110 (45)	176 (46)	176 (43)	
Liquids only	75 (5)	2 (5)	8 (5)	13 (5)	14 (6)	13 (3)	25 (6)	
Soft or liquid food only	290 (20)	11 (27)	28 (16)	51 (21)	41 (17)	78 (21)	81 (20)	
Total dysphagia	22 (1)	1 (2)	3 (2)	5 (2)	2 (1)	5 (1)	6 (1)	
Odynophagia	287 (19)	10 (24)	45 (26)	57 (24)	50 (20)	77 (20)	48 (12)	< 0.001
Nausea	133 (9)	5 (12)	21 (12)	29 (12)	14 (6)	32 (8)	32 (8)	0.082
Jaundice	4 (0)	0 (0)	0 (0)	1 (0)	1 (0)	2 (1)	0 (0)	0.703
Hepatomegaly	5 (0)	0 (0)	1 (1)	3 (1)	0 (0)	1 (0)	0 (0)	0.121
Anemia	102 (7)	0 (0)	12 (7)	16 (7)	15 (6)	20 (5)	39 (10)	0.098
Reflux	376 (25)	7 (17)	69 (41)	45 (19)	48 (20)	105 (28)	102 (25)	< 0.001

Data are expressed as *n* (%) unless otherwise specified

*IQR* interquartile range, *SCC* squamous cell carcinoma, *BMI* body mass index, *ASA* American Society of Anesthesiologists

### Changes in Staging

Over the study period, a gradual evolution of staging investigations was employed. The routine use of bone scans was phased out and PET/CT scans have become standard for all patients being considered for curative treatment. Endoscopic ultrasound is now also routinely performed to help evaluate the depth of tumor invasion and local lymph node involvement. Those who underwent surgery were increasingly at a more advanced clinical stage (stage III/IV), with 74% of patients in the earlier cohort having stage 2 disease or lower, compared with 20% in the final cohort (*p* < 0.001).

### Changes in Pathology

Median lymph node yield was significantly lower in the first two cohorts (10 & 19 nodes) compared with the last three cohorts (30–34 nodes, *p* < 0.001), although longitudinal R0 resection rates were consistently under 5%^19^ (Table 2 and “Appendix 2”).

**Table 2:** Histopathological outcomes of patients having esophagectomy

	Overall	1989–1993	1994–1998	1995–2003	2003–2008	2009–2013	2014–2018	*p* value
*n*	1486	41	170	240	245	380	410	
Overall clinical stage								< 0.001
I	249 (17)	27 (66)	54 (32)	22 (9)	35 (14)	47 (12)	64 (16)	
IIA	102 (7)	5 (12)	26 (15)	18 (8)	12 (5)	16 (4)	25 (6)	
IIB	68 (5)	1 (2)	8 (5)	16 (7)	12 (5)	9 (2)	22 (5)	
III	835 (56)	6 (15)	74 (44)	180 (75)	182 (74)	206 (54)	187 (46)	
IVA	224 (15)	2 (5)	8 (5)	3 (1)	3 (1)	99 (26)	109 (27)	
IVB	8 (1)	0 (0)	0 (0)	1 (0)	1 (0)	3 (1)	3 (1)	
Overall pathological stage								< 0.001
0/I	84 (6)	0 (0)	6 (4)	9 (4)	12 (5)	12 (3)	45 (11)	
IA	85 (6)	0 (0)	15 (9)	26 (11)	20 (8)	11 (3)	13 (3)	
IB	162 (11)	6 (15)	10 (6)	17 (7)	22 (9)	46 (12)	61 (15)	
IC	76 (5)	1 (2)	4 (2)	10 (4)	14 (6)	26 (7)	21 (5)	
IIA	92 (6)	3 (7)	13 (8)	8 (3)	12 (5)	30 (8)	26 (6)	
IIB	226 (15)	6 (15)	16 (9)	31 (13)	46 (19)	68 (18)	59 (14)	
IIIA	122 (8)	8 (20)	20 (12)	19 (8)	23 (9)	23 (6)	29 (7)	
IIIB	506 (34)	17 (41)	79 (46)	106 (44)	87 (36)	111 (29)	106 (26)	
IVA	122 (8)	0 (0)	6 (4)	12 (5)	8 (3)	49 (13)	47 (11)	
IVB	6 (0)	0 (0)	1 (1)	2 (1)	0 (0)	2 (1)	1 (0)	
Unknown	5 (0)	0 (0)	0 (0)	0 (0)	1 (0)	2 (1)	2 (0)	
Tumor grade								< 0.001
Well-differentiated	136 (9)	7 (17)	29 (17)	32 (13)	30 (12)	16 (4)	22 (5)	
Moderately differentiated	683 (46)	15 (37)	61 (36)	123 (51)	108 (44)	198 (52)	178 (43)	
Poorly differentiated	516 (35)	14 (34)	66 (39)	67 (28)	84 (34)	140 (37)	145 (35)	
Unknown	151 (10)	5 (12)	14 (8)	18 (8)	23 (9)	26 (7)	65 (16)	
Lymph nodes examined (median [IQR])	29 [21–39]	10 [7–13]	19 [13–24]	27 [19–34]	34 [26–42]	30 [23–38]	34 [27–45]	< 0.001
Lymph nodes positive (median [IQR])	1 [0–4]	1 [0–4]	1 [0–5]	1 [0–5]	1 [0–4]	1 [0–3]	0 [0–3]	0.001
Proximal margin R1	9 (1)	0 (0)	0 (0)	0 (0)	2 (1)	6 (2)	1 (0)	0.081
Distal margin R1	13 (1)	0 (0)	0 (0)	0 (0)	1 (0)	1 (0)	11 (3)	0.001
Lymphatic invasion	635 (43)	3 (7)	59 (35)	110 (46)	116 (47)	186 (49)	161 (39)	< 0.001
Venous invasion	476 (32)	2 (5)	39 (23)	77 (32)	83 (34)	154 (41)	121 (30)	< 0.001
Perineural invasion	626 (42)	14 (34)	68 (40)	125 (52)	115 (47)	162 (43)	142 (35)	< 0.001
Extracapsular spread	225 (15)	0 (0)	0 (0)	0 (0)	25 (10)	105 (28)	95 (23)	< 0.001

Data are expressed as *n* (%) unless otherwise specified

*IQR* interquartile range

### Patient Outcomes

Overall median survival for the entire cohort was 29 months (IQR 15–68 months). There was a fall in in-patient mortality, from 5 to 2%, between the first and last cohorts (*p* = 0.002). Median survival increased between each cohort, from 22.1 months in the first cohort to 56.0 months in the final cohort (*p* < 0.001). Overall complications were 65%, which increased over the time period and were initially 54% through to 68% in the latest cohort (*p* = 0.004). However, there were no significant differences in the anastomotic leak rate between the time cohorts, varying from 7 to 11% (*p* = 0.6) [Table 3].

**Table 3:** Trends in operative management and outcomes

	Overall	1989–1993	1994–1998	1995–2003	2003–2008	2009–2013	2014–2018	*p* value
*n*	1486	41	170	240	245	380	410	
Operation type (%)								< 0.001
Ivor Lewis	1429 (96)	39 (95)	163 (96)	237 (99)	237 (97)	367 (97)	386 (94)	
Left thoraco-abdominal	15 (1)	2 (5)	2 (1)	3 (1)	5 (2)	2 (1)	1 (0)	
McKeown	38 (3)	0 (0)	1 (1)	0 (0)	3 (1)	11 (3)	23 (6)	
Transhiatal	4 (0)	0 (0)	4 (2)	0 (0)	0 (0)	0 (0)	0 (0)	
Unimodality surgery	754 (51)	41 (100)	168 (99)	201 (84)	105 (43)	119 (31)	120 (29)	< 0.001
Surgical access thoracic phase								
Open	1431 (96)	41 (100)	170 (100)	240 (100)	245 (100)	376 (99)	359 (88)	
Thoracoscopic	46 (3)	0 (0)	0 (0)	0 (0)	0 (0)	0 (0)	46 (11)	
Thoracoscopic converted	4 (0)	0 (0)	0 (0)	0 (0)	0 (0)	0 (0)	4 (1)	
Surgical access abdominal phase								
Open	1468 (99)	41 (100)	170 (100)	240 (100)	245 (100)	363 (96)	409 (100)	
Laparoscopic	12 (1)	0 (0)	0 (0)	0 (0)	0 (0)	12 (3)	0 (0)	
Laparoscopic converted	6 (0)	0 (0)	0 (0)	0 (0)	0 (0)	5 (1)	1 (0)	
Critical care stay (median [IQR])	2 [1–5]	2 [1–3]	1 [1–3]	3 [1–8]	2 [1–5]	3 [1–5]	3 [2–5]	< 0.001
Length of stay (median [IQR])	15 [12–22]	14 [12–18]	13 [12–16]	17 [14–27]	16 [14–25]	16 [13–25]	11 [8–18]	< 0.001
Overall complications	963 (65)	22 (54)	93 (55)	172 (72)	159 (65)	239 (63)	278 (68)	0.004
Surgical site infection	130 (9)	0 (0)	8 (5)	33 (14)	30 (12)	27 (7)	32 (8)	0.001
Pulmonary complications	188 (13)	1 (2)	3 (2)	9 (4)	11 (4)	72 (19)	92 (22)	< 0.001
Cardiac complications	107 (7)	0 (0)	1 (1)	2 (1)	2 (1)	40 (11)	62 (15)	< 0.001
Anastomotic leaks	126 (8)	3 (7)	10 (6)	17 (7)	26 (11)	33 (9)	37 (9)	0.584
In-hospital mortality	58 (4)	2 (5)	11 (6)	15 (6)	10 (4)	7 (2)	13 (3)	0.042
30-day mortality	43 (3)	2 (5)	10 (6)	13 (5)	6 (2)	2 (1)	10 (2)	0.002
90-day mortality	69 (5)	3 (7)	15 (9)	16 (7)	15 (6)	8 (2)	12 (3)	0.001

Data are expressed as *n* (%) unless otherwise specified

*IQR* interquartile range

### Patients Undergoing Thoracoscopic Surgery

A thoracoscopic program commenced in October 2014. Over this time period, 50 patients underwent thoracoscopic procedures, with four conversions to an open thoracotomy. The median age of patients was 66 years (IQR 60–71), 79% were male, and 20% were squamous cell carcinomas. There was no mortality in this cohort and the median length of stay was 8 days (versus 11 days for open thoracotomy; *p* < 0.001), with an overall complication rate of 66%. Median lymph node yield was 36 (IQR 27–42), with no R1 resections recorded.

### Patients Undergoing Endoscopic Mucosal Resection

Overall, 197 EMRs were undertaken in 157 patients with curative intent. The median age of patients was 70 years (IQR 61–78). The initial EMR was undertaken in 2007; however, only 13 patients underwent EMR between 2007 and 2011. The final 5-year cohort included 127 patients. Of the 157 patients, 43 (27%) subsequently underwent esophagectomy; there have been no deaths in any of these 43 patients.

### Notable Interventions

A number of notable interventions occurred during the time frame of this study. These are highlighted in Fig. 1, which demonstrates the trend in the number of cases performed and also demonstrates the relative impact on 5-year survival, length of stay, and the proportion of patients with stage III and IV disease.

**Fig. 2:**
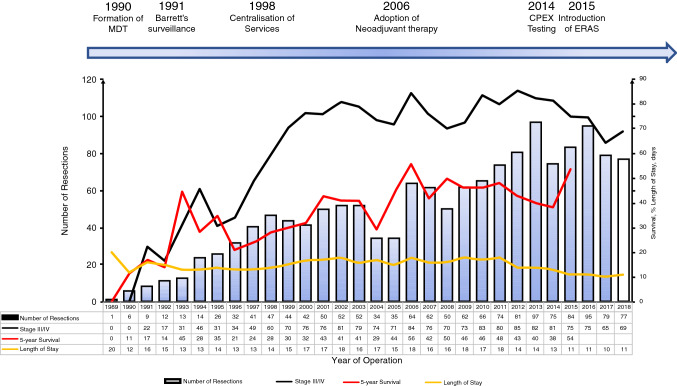
Evolution in number of cases, survival, and length of stay correlated with major interventions. Bars demonstrate the number of cases each year. *MDT* multidisciplinary team, *ERAS* enhanced recovery after surgery, *CPEX* cardiopulmonary exercise testing

 The major interventions included are (Fig. 2):

1. Institution of MDT meetings 1990
2. Barrett’s surveillance program, 1991
3. Centralization of cancer work, 1998
4. Routine neoadjuvant treatment, 2006
5. Cardiopulmonary exercise testing (CPET as standard, 2014
6. Routine enhanced recovery, 2015
7. Introduction of prehabilitation, 2018

## Discussion

The results from this study highlight the changing trend in presentation, management, and outcomes of patients with esophageal cancer. Perhaps the most noteworthy, but not unexpected, finding is the considerable improvement in patient survival over the last 30 years. Median survival for all patients undergoing surgery is now 52 months, reflecting a 5-year survival of just under 50%—nearly double what it was in the early part of the study period. The reasons for this are likely to be multifactorial, but the largest impact is probably due to the establishment of neoadjuvant treatment for patients with locally advanced disease. Patients first received neoadjuvant treatment in 1998, and subsequent cohorts where the use of neoadjuvant treatment became the standard for locally advanced cancer demonstrated a significant jump in survival from the earlier cohorts. The OE02 study showed a 6% 5-year survival benefit associated with neoadjuvant chemotherapy when compared with surgery alone.^6^ These findings were supported by the UK MAGIC study, which reported a 13% improvement in 5-year survival associated with the use of perioperative chemotherapy in esophagogastric cancer.^5^

In addition, this study showed that in-hospital mortality also fell from an initial 5% to 2%, which may reflect increased experience in managing postoperative complications.^20^,^21^ The UK national audit has placed the current mortality from esophagectomy at approximately 3%, but this is a dramatic reduction in mortality compared with what was reported towards the end of the last century.^22^,^23^ Further to the improved survival was a reduction in the length of stay in hospital over the time period. This was most noticeable in the final cohort, which coincides with the institution of a standardized enhanced recovery program. There was no change in the median length of critical care stay over the 30-year period. The data do not contain information as to why patients may have a prolong critical care stay, with there likely being multiple reasons, including patient need (e.g. vasopressor support), availability of a stepdown level 1 ward bed, or a feeling that prolong observation was required in critical care due to comobidities. It is worth nothing that prior to 2001, patients were routine returned to critical care intubated post procedure, with planned extubation the following day. However, this change of practice has not impacted on overall critical care stay, although it will have significantly reduced the need for level 3 care and increased the use of level 2 beds.

To contrast this, there has been a steady increase in the overall reported incidence of complications, which, in the latest cohort, was 71%. The reported postoperative complications in the literature vary at between 40 and 75%, which is consistent with what was found throughout each of the time periods. Possibly the two main reasons for the increased level of complications are more assiduous reporting and an increased diagnosis of patients with ‘curable’ disease but a higher level of comorbidity. This is reflected in the increased BMI of patients over the time period and worse American Society of Anesthesiologists (ASA) score of those undergoing surgery. Despite this, the results demonstrate that a standardized surgical approach, careful consideration of the patient pathway, which saw the introduction of multiple components, including neoadjuvant treatment for locally advanced disease, CPET, and the institution of an enhanced recovery after surgery (ERAS) program, can lead to improved patient outcomes.^21^,^24^,^25^ However, it is important to constantly evaluate current practices. Such an ethos led to the acceptance by the clinical team that multimodal analgesia provides excellent outcomes for patients.

Another recent intervention has been the increasing use of thoracoscopic surgery. This was implemented in the final cohort, with the ethos of maintaining the same level of lymphadenectomy as performed with an open operation. Within this final time frame, 50 patients underwent a thoracoscopic chest phase, with only four (8%) requiring conversion to an open procedure. The overall median length of stay was 8 days, which compared favorably with the open cohort, with a similar complication rate (66%) and no mortality.

A further notable finding was the change in frequency of presenting symptoms. Nearly two-thirds of patients now state abdominal discomfort as a presenting symptom compared with under 20% at the end of the last century. This might highlight an increasing awareness of the implication of epigastric symptoms among primary care physicians, or increased inclination to investigate these symptoms at an earlier stage. A previous study indicated that only carrying out endoscopy in dyspeptics with ‘alarm’ symptoms would overlook a significant proportion of patients with a malignancy.^26^ Thus, better access to endoscopic evaluation may have contributed to patients being picked up at a potentially curable stage, and may in turn contribute to the steady increase in the number of operations performed each year. The future may present methods to aid screening for these cancers, such as a cytosponge.^27^

Despite the increased number of operations that have occurred each year, there was a negligible increase in the number of patients with stage 1 disease who underwent surgery. The recognition that an EMR can achieve good oncological outcomes for those with early disease confined to the mucosa, where lymph node metastasis is yet to occur, has meant that fewer patients have needed to be subjected to the morbidity of an esophagectomy. The first EMR was performed in 2007; however, only a small number of patients underwent this procedure in the first few years, and this became established as an intervention within the final cohort, with 127 patients having an EMR with the intention to treat cancer. While an EMR can be used to cure early cancers (T1a), debate remains regarding its use for more advanced (T1b) cancers. It is also possible to employ this as a treatment for those who may be regarded as too frail for a more invasive esophagectomy. Importantly, none of the patients who underwent an EMR and subsequently had an esophagectomy have died, suggesting that an EMR does not compromise patient outcomes, although the introduction of an EMR is likely to have influenced the overall number of procedures that have been performed and also the number of patients with an early-stage cancer.

The major shortcoming of this study, which could also be argued as being its main strength, is that data have come from a single center with consistent reporting over time. It highlights the changes that have occurred in esophageal cancer treatment and the benefits of standardization of care. Over the 30-year period, 12 surgeons have been responsible for performing resections. The latest cohort included a small number of patients who underwent a thoracoscopic chest phase, but, despite this, perioperative management and surgical dissection and reconstruction were consistent with those having open surgery. Other deficits include a lack of data on comorbidities, with the ASA score used as a surrogate within the study. Arguably one of the most important factors that this paper does not address is postoperative quality of life. This should be a key consideration, especially when minimally invasive and robotic techniques are being considered for patients. While some of the changes have been highlighted throughout this paper, this list is unlikely to be exhaustive and it is impossible to quantify the impact of changes in nutritional assessment and management within the critical care environment, and perioperatively, that may have contributed towards the improved outcome. It is also impossible to fully quantify the impact of the changes that have been implemented due to these confounding factors.

A number of fundamental changes to the management of esophageal cancer patients have been integrated that will affect patients, subsequent to those included in this study. Prehabilitation has been shown to aid patients in maintaining fitness during neoadjuvant chemotherapy, and a pragmatic approach that is accessible to all patients has been instituted. The recently published FLOT trial has indicated a significant survival improvement for patients and this may further enhance the outcomes seen.^28^ The ability to identify biomarkers for prognostication and tailoring of treatment will surely ensue over the next 30 years, as might methods for screening for esophageal cancer, allowing more patients to be identified at earlier stages.

From a surgical perspective, minimally invasive techniques and robotic technology may help reduce morbidity.^29^ However, throughout this study, patients had a radical lymphadenectomy, which has been demonstrated to improve outcomes, and care must be taken to observe oncological principles.^30^,^31^ The standardization of reporting outcomes and complications will help facilitate meaningful comparison of data between units and the production of robust studies to improve outcomes.^32^ It may be that technology such as indocyanine green-enhanced fluorescence can improve anastomotic outcomes,^33^,^34^ or that an improved method of identifying lymph node involvement can help tailor the extent of lymphadenectomy required,^35^–^37^ given previous studies have highlighted relative unpredictability in this area.^38^,^39^

## Conclusions

This study highlights the continual evolution in the treatment and outcomes of patients with esophageal cancer. There is ever-increasing research to allow tailoring of oncological therapies for the improvement of patient outcomes, and these results have indicated that a consistent surgical approach that has been adopted by all team members provides excellent surgical outcomes. Adjuncts that aid decision making, improve the fitness of patients, and enable better postoperative rehabilitation need constant evaluation so they can be appropriately integrated to further enhance outcomes. These results serve to highlight areas where little progress has been made over the last 30 years. Rates of morbidity, including anastomotic leak and pulmonary complications, are areas that need to be focused on in the future.

## Appendix 1: Univariable and Multivariable Analysis of Impact on Survival

See Table 4.

**Table 4:** Univariable and multivariable analysis of factors impacting on survival

		HR (univariable)	HR (multivariable)
Year25		–	
	1989–1993	1.61 (0.98–2.63, *p* = 0.058)	1.16 (0.58–2.30, *p* = 0.675)
	1994–1998	1.71 (1.14–2.56, *p* = 0.009)	1.49 (0.90–2.47, *p* = 0.123)
	1999–2003	1.40 (0.94–2.08, *p* = 0.099)	1.25 (0.78–2.00, *p* = 0.350)
	2004–2008	1.13 (0.76–1.69, *p* = 0.536)	1.08 (0.68–1.71, *p* = 0.734)
	2009–2013	1.14 (0.77–1.69, *p* = 0.509)	1.05 (0.69–1.59, *p* = 0.828)
	2014–2018	0.91 (0.60–1.38, *p* = 0.665)	0.93 (0.60–1.43, *p* = 0.727)
Age at presentation	Mean (SD)	1.02 (1.01–1.02, *p* < 0.001)	1.02 (1.01–1.02, *p* < 0.001)
Gender	Male	–	–
	Female	0.80 (0.69–0.94, *p* = 0.005)	0.72 (0.60–0.87, *p* = 0.001)
Histology	Adenocarcinoma	0.99 (0.85–1.14, *p* = 0.853)	0.71 (0.58–0.86, *p* = 0.001)
	SCC	–	–
BMI	Mean (SD)	0.97 (0.95–0.98, *p* < 0.001)	0.98 (0.97–1.00, *p* = 0.023)
Smoking status	Current	–	–
	Ex-Smoker	0.84 (0.72–0.98, *p* = 0.027)	0.93 (0.78–1.11, *p* = 0.405)
	Never	0.74 (0.63–0.88, *p* = 0.001)	0.88 (0.72–1.07, *p* = 0.201)
	Unknown	1.00 (0.55–1.83, *p* = 0.993)	0.86 (0.35–2.15, *p* = 0.751)
ASA grade	Grade 1	–	–
	Grade 2	1.17 (0.96–1.42, *p* = 0.113)	1.22 (0.98–1.51, *p* = 0.070)
	Grade 3	1.40 (1.13–1.73, *p* = 0.002)	1.29 (1.01–1.64, *p* = 0.040)
	Grade 4	2.04 (1.00–4.17, *p* = 0.049)	1.81 (0.83–3.97, *p* = 0.137)
	Unknown	1.28 (1.00–1.65, *p* = 0.050)	0.97 (0.72–1.31, *p* = 0.864)
Overall treatment2	NAC + Surgery	0.86 (0.76–0.99, *p* = 0.030)	0.87 (0.71–1.08, *p* = 0.213)
	Surgery Only	–	–
Pathological stage of disease	Stage 0/I	–	–
	Stage IA	0.97 (0.60–1.55, *p* = 0.886)	1.55 (0.81–2.95, *p* = 0.184)
	Stage IB	0.83 (0.52–1.30, *p* = 0.406)	1.21 (0.67–2.20, *p* = 0.525)
	Stage IC	1.23 (0.75–2.00, *p* = 0.411)	1.7 (0.91–3.17, *p* = 0.094)
	Stage IIA	1.03 (0.63–1.68, *p* = 0.897)	1.2 (0.64–2.24, *p* = 0.574)
	Stage IIB	1.28 (0.85–1.92, *p* = 0.244)	1.42 (0.81–2.47, *p* = 0.220)
	Stage IIIA	2.57 (1.69–3.90, *p* < 0.001)	2.35 (1.35–4.08, *p* = 0.003)
	Stage IIIB	3.98 (2.73–5.81, *p* < 0.001)	3.02 (1.74–5.24, *p* < 0.001)
	Stage IVA	5.69 (3.75–8.61, *p* < 0.001)	3.1 (1.69–5.68, *p* < 0.001)
	Stage IVB	10.83 (4.48–26.20, *p* < 0.001)	5.02 (1.42–17.73, *p* = 0.012)
	Unknown	16.46 (6.33–42.77, *p* < 0.001)	–
Tumour_GRade	Well	–	–
	Moderate	1.63 (1.27–2.08, *p* < 0.001)	1.27 (0.89–1.82, *p* = 0.188)
	Poor	2.39 (1.86–3.07, *p* < 0.001)	1.42 (0.98–2.03, *p* = 0.061)
	Unknown	1.51 (1.10–2.09, *p* = 0.011)	1.47 (0.95–2.28, *p* = 0.087)
Lymph nodes examined	Mean (SD)	1.00 (0.99–1.00, *p* = 0.074)	0.99 (0.99–1.00, *p* = 0.041)
Positive lymph nodes	Mean (SD)	1.12 (1.11–1.13, *p* < 0.001)	1.06 (1.04–1.08, *p* < 0.001)
Longitudinal Margin	R0	–	–
	R1	4.39 (3.04–6.36, *p* < 0.001)	2.03 (1.15–3.57, *p* = 0.014)
Lymph involvement	No	–	–
	Yes	2.19 (1.92–2.49, *p* < 0.001)	1.25 (1.04–1.51, *p* = 0.020)
Venous involvement	No	–	–
	Yes	1.98 (1.73–2.26, *p* < 0.001)	0.92 (0.76–1.11, *p* = 0.383)
Perineural involvement	No	–	–
	Yes	2.51 (2.20–2.86, *p* < 0.001)	1.36 (1.13–1.64, *p* = 0.001)
Tumour regression grade	1	–	–
	2	1.04 (0.46–2.37, *p* = 0.929)	0.93 (0.39–2.18, *p* = 0.862)
	3	1.40 (0.74–2.63, *p* = 0.302)	0.99 (0.50–1.94, *p* = 0.971)
	4	2.87 (1.62–5.08, *p* < 0.001)	1.18 (0.64–2.20, *p* = 0.591)
	5	3.61 (1.92–6.79, *p* < 0.001)	1.06 (0.53–2.11, *p* = 0.863)
	Unknown	2.61 (1.50–4.52, *p* = 0.001)	1.23 (0.68–2.23, *p* = 0.490)
Extracapsular spread	No	–	–
	Yes	2.39 (2.02–2.82, *p* < 0.001)	1.72 (1.37–2.17, *p* < 0.001)

## Appendix 2: Univariable and Multivariable Analysis of Impact on Survival

See Table 5.

**Table 5:** Distribution of TNM stage for patients that had neoadjuvant chemotherapy and surgery and those that had unimodality surgery

	N0	N1	N2	N3
Neoadjuvant and surgery				
T0	51	6	2	0
T1	66	27	3	0
T2	72	55	9	2
T3	115	157	73	51
T4	9	15	9	10
Surgery only				
T0	25	1	0	0
T1	217	25	1	2
T2	41	43	3	0
T3	79	253	19	13
T4	10	16	2	4
